# Current Concepts in Platelet-Rich Plasma Therapy for Lateral Epicondylitis Through a Histopathological and Clinical Lens

**DOI:** 10.7759/cureus.110311

**Published:** 2026-06-05

**Authors:** Navid R Reshadi Nezhad, Meghan McCullough

**Affiliations:** 1 School of Medicine, California University of Science and Medicine, Colton, USA; 2 Department of Plastic Surgery, Cedars-Sinai Medical Center, Los Angeles, USA

**Keywords:** biologic treatment, elbow joint, forearm pain, lateral epicondylitis elbow, tendon healing

## Abstract

Lateral epicondylitis (LE), or tennis elbow, is a degenerative tendinopathy of the extensor carpi radialis brevis (ECRB) tendon. While most patients improve with conservative therapies, a subset experience chronic, refractory symptoms requiring advanced interventions. Platelet-rich plasma (PRP) has emerged as a promising biologic treatment, offering an autologous source of growth factors that may enhance tendon healing. This review examines current concepts in PRP therapy for LE, including basic science mechanisms, animal studies, and clinical outcomes.

Chronic LE is characterized by a paucity of inflammatory infiltrate and increased programmed cell death (PCD), suggesting a limited intrinsic healing response. PRP delivers key growth factors (vascular endothelial growth factor (VEGF), platelet-derived growth factor (PDGF), insulin-like growth factor (IGF), fibroblast growth factor (FGF), and transforming growth factor-beta (TGF-β)), each of which has demonstrated pro-healing properties in animal models by promoting angiogenesis, fibroblast proliferation, and collagen remodeling. Clinical data on PRP are heterogeneous, with some randomized trials and long-term follow-up studies demonstrating significant improvement in pain and function, while others show minimal benefit. Comparisons with corticosteroids suggest that PRP may offer superior long-term outcomes, although corticosteroids provide better short-term relief. PRP has also shown comparable efficacy to surgical debridement, offering a less invasive alternative.

The role of leukocyte concentration in PRP remains unresolved; while leukocyte-poor PRP may reduce fibrosis and inflammation, clinical studies have not definitively favored one formulation over another. Overall, PRP therapy represents a biologically plausible, relatively safe, and potentially effective treatment option for refractory LE. Further research is needed to optimize formulation, delivery, and patient selection to fully realize its clinical utility.

## Introduction and background

Lateral epicondylitis (LE), colloquially known as tennis elbow, is among the most common causes of elbow pain. Frequently seen in racquet sports athletes, LE is strongly associated with repeated wrist extension and hand gripping, both of which can lead to degeneration of the extensor carpi radialis brevis (ECRB) tendon at the lateral epicondyle of the humerus. In most patients, nonoperative treatments suffice to improve pain and reduce debilitation. These include activity modification, red light therapy, physiotherapy, nonsteroidal anti-inflammatory medications, bracing, acupuncture, extracorporeal shock therapy, and corticosteroids [1]. However, for patients with refractory pain, further intervention is required. Although operative intervention and steroid injection are classically indicated, the examination of platelet-rich plasma (PRP) treatment as a replacement therapy is warranted, as it has shown promise in both animal and human studies. The present narrative review will provide a histopathological basis for PRP efficacy, which may provide a mechanistic basis for clinical PRP efficacy.

## Review

Lateral epicondylitis pathogenesis

The exact pathogenic mechanism of chronic LE is not known. It has classically been the consensus that LE is an inflammatory condition. However, advances in our understanding of chronic LE pathogenesis point to a degenerative, not inflammatory, etiology [2]. Initially, in acute LE, there is an influx of inflammatory infiltrate and increased release of platelet granule growth factors, notably vascular endothelial growth factor (VEGF), platelet-derived growth factor (PDGF), insulin-like growth factor (IGF), fibroblast growth factor (FGF), and transforming growth factor-beta (TGF-β) [3-5]. These growth factors induce mesenchymal stem cell differentiation, chondrocyte proliferation, osteoblastogenesis, intratendinous angiogenesis, and chondrocyte apoptosis inhibition, promoting tendon healing [6]. However, this initial inflammatory response wanes over time, such that in patients with refractory LE, there is a marked reduction of inflammatory infiltrate. Regan et al. found that polymorphonuclear leukocytes, lymphocytes, and lipid-laden macrophages were virtually absent from chronic LE surgical specimens [7]. Furthermore, Alfredson et al. found no difference in ECRB tendon prostaglandin E2 (PGE2) levels between patients with LE and controls, strongly suggesting that LE is a tendinosis, not a tendinitis [8].

Aside from the reduction in inflammatory infiltrate, there is a reduction in overall cellularity within the ECRB tendon in LE. This is attributed to an increase in programmed cell death (PCD). It has been shown that the degree of autophagy and apoptosis is positively correlated with both the extent of ECRB damage and the grade of collagen damage. Thus, PCD appears to be implicated in the pathogenesis of refractory LE, as reduced cellularity may blunt the healing capacity of the damaged tendon [9]. Programmed cell death constitutes a vital part of Nirschl et al.’s characterization of LE pathologic stages in response to ECRB damage. The first stage involves initial inflammation. The second stage describes pro-healing fibroblastic and vascular hyperplasia that results in disorganized collagen deposition. In the absence of repeated damage to the ECRB tendon, long-term collagen remodeling is expected to restore the tendon’s tensile strength. However, persistent overuse of the injured tendon induces significant PCD, reducing the number of pro-healing cells and, thus, eventually exhausting the healing response. In the third stage, as cellularity in the tendon falls, its structural integrity declines until either structural failure or rupture occurs. The final stage is characterized by fibrosis and calcification of the damaged tendon [2].

Platelet alpha granule growth factor (VEGF, PDGF, IGF, FGF, and TGF-β) studies in animals

Our knowledge of platelet-rich plasma (PRP) treatment efficacy for lateral epicondylitis is limited due to the nascency of this treatment modality. However, emerging studies in animals, while utilizing imperfect simulations of human LE, suggest a beneficial effect of this treatment. PRP is an autologous blood product consisting of plasma with a high concentration of platelets. Upon injection, platelet adhesion within the injury site activates growth factor release from alpha granules [6]. It is posited that the growth factors contained within platelet alpha granules, notably VEGF, PDGF, IGF, FGF, and TGF-β, are responsible for the potential beneficial effect of PRP treatment in tendinopathy. The efficacy of these growth factors in animal tendon healing will be examined individually in the following section. It should be noted that autologous blood injection, an alternative to concentrated, platelet-rich plasma, is a potential treatment option. In a meta-analysis, Arirachakaran et al. found that while both autologous blood and PRP injection improved pain and disability scores, the effect of PRP was significantly greater than that of autologous blood. Additionally, autologous blood injection was associated with a higher risk of adverse effects [10]. While autologous blood injection appears to be a viable treatment option to reduce pain, PRP is preferred for its reduced risk of adverse reaction and, more importantly, higher concentration of platelets, with the presumption that the beneficial effect of both autologous blood and PRP injection is derived from contents within platelet alpha granules.

Vascular Endothelial Growth Factor (VEGF)

VEGF is a potent pro-angiogenic growth factor found in platelet granules. It promotes angiogenesis through the induction of endothelial cell migration and proliferation and inhibition of apoptosis [11]. VEGF-induced angiogenesis has been shown to be a vital part of tendon healing in rats; considering the key role of VEGF in physiological angiogenesis, it is thought that VEGF administration into injured tendon tissue may increase vascularity and subsequent access of pro-healing cells to the injury site, promoting healing [12].

The impact of local VEGF injection on the structural integrity of injured tendons has been examined in several studies, which will be examined here. One study involved surgical resection of rat Achilles tendon proximal to its calcaneal insertion and injection of VEGF-111, a recombinant VEGF-A isoform, into the injury site. While ultimate tensile strength (UTS) increased by day 30 post-surgery in the control group, VEGF administration led to a greater increase in UTS than that seen in purely physiological healing [5]. In a study with a similar tendon injury and VEGF injection protocol in rats, it was shown that VEGF administration into the injury site significantly increases oxygenation by day 14 post-surgery, more than in controls. This increase in oxygenation occurs concurrently with the increase in maximum load tolerated by the tendon as compared to controls. Upon histological examination of tendon specimens, there was no increase in collagen organization; the authors postulated changes in collagen content and altered composition of non-collagen extracellular matrix (ECM) components to be responsible for the increase in tendon structural integrity observed after VEGF injection [13]. In another study, Zhang et al. found that VEGF injection into injured rat Achilles tendon led to increased tensile strength by week 2 postoperatively compared to controls. However, four weeks post-surgery, there was no significant difference in tensile strength among the groups [14]. This finding suggests that VEGF is involved early on in the tendon healing process.

Despite its posited role in pro-healing angiogenesis, it should be noted that VEGF may exacerbate tendon ECM degeneration in chronic LE through its induction of matrix metalloproteinase expression and inhibition of metalloproteinase inhibitors (TIMPs) [15].

Platelet-Derived Growth Factor (PDGF)

Platelet-derived growth factor (PDGF) is thought to promote tendon healing through its mitogenic and chemotactic stimulation of tendon cells within and around the tendon injury site [3,16]. This may explain the finding that PDGF has been shown to reduce scar formation in rat Achilles tendon recovery, potentially improving the structural integrity of regenerating tissue in chronic LE [17]. Further, it has been shown that inactivation of platelet-derived growth factor receptor alpha (Pdgfra) blocks tenocyte proliferation and tendon regeneration, suggesting a key role of PDGF in tendon healing [18]. Thus, PDGF may provide the signaling needed to promote healing in LE.

One study in rat Achilles tendon injury found significantly improved scores of vascularity, cellularity, fiber parallel, and fiber diameter after PDGF microneedle systemic administration. The authors hypothesize this improvement in the ECM environment to be induced by PDGF chemotactic recruitment of tenocytes and fibroblasts to the injury site. These results may explain the increase in maximum load, stiffness, and stress of PDGF-treated tendons compared to controls [3]. A methodologically similar study done in rabbits found a significant increase in mature tenocyte density within injury sites of Achilles tendons treated with PDGF at week 3 post-operation. PDGF also decreased fibrosis, as indicated by a decrease in alpha-SMA-positive cells (a marker of fibrosis) [17]. Additionally, several studies demonstrated a PDGF-induced increase in collagen type I and proteoglycan synthesis and a decrease in type III collagen synthesis in horse and rabbit tendon injury [16,19].

These findings suggest a pro-healing role of PDGF in tendon injury, which seems to entail the enhancement of anti-fibrotic tendon healing through recruitment of tenocytes to the injury area. The data is not conclusive, however. Despite these positive findings, Kovacevic et al. found no significant difference in rat rotator cuff maximum tensile strength between the PDGF group and controls four weeks post-surgery [20].

Insulin-Like Growth Factor (IGF)

Insulin-like growth factor (IGF) is posited to be a stimulus for fibroblast proliferation and maturation, DNA synthesis, and collagen I-dominant extracellular matrix synthesis [21]. Further, it has been shown to have an anti-apoptotic effect on fibroblasts, which may provide a beneficial effect in LE, considering that LE pathogenesis is associated with increased programmed cell death within the injured tendon [22]. Chen et al. found that IGF-1 increased in acute tendon injury and stayed elevated throughout most phases of tendon healing, suggesting an important role in promoting healing through the maintenance of fibroblast and tenocyte proliferation and ECM repair [23]. Additionally, a synergistic optimal effect of IGF-I and PDGF on tendon fibroblast proliferation has been demonstrated, a pertinent finding considering that PRP treatment involves the conjoined administration of these growth factors in addition to other pro-healing growth factors and compounds [24].

Regarding the in vivo effect of IGF activity, Wu et al. found that the activation of IGFIR signaling, via ginsenoside Rg1 administration, led to a dose-dependent increase in injured rat Achilles tendon tensile strength and stiffness compared to controls. Furthermore, it was found that Rg1 administration, through its activation of IGFIR, enhanced collagen fiber organization at week 8 post-surgery, suggesting a role of IGF in late-stage collagen remodeling [25]. It should be noted, however, that this study involved indirect IGF administration and thus may not reliably model the potential effect of direct IGF administration. In a rat rotator cuff injury model, IGF-infused matrix implantation into the injury site led to increased supraspinatus tendon failure load, strength, toughness, and stiffness eight weeks post-surgery. On histological examination, the IGF group was observed to have an improved histological score with greater collagen organization than controls, which may account for the increase in structural integrity demonstrated [26].

Despite these findings, an equine flexor digitorum profundus (FDP) tendon injury study found no significant difference in ultimate failure load and stiffness of FDP tendon between the IGF-I treatment group and controls. Furthermore, there was no difference in histological score found between the groups [27].

Fibroblast Growth Factor (FGF)

Fibroblast growth factor (FGF) has been shown to be involved in mesenchymal stem cell and chondrocyte differentiation and proliferation [28,29]. Furthermore, Chen et al. found FGF to be a prognostic marker of one-year patient outcome after surgical repair of ruptured Achilles tendon; increased FGF levels were associated with improved Achilles Tendon Total Rupture Score and decreased pain rating and debilitation [30]. Thus, FGF appears to be associated with tendon healing. FGF may enhance healing by increasing fibroblastic cellularity at the injury site, which has been established to be dramatically decreased in ECRB tendons of patients with refractory LE [9].

Yonemitsu et al. analyzed the effect of FGF-2 on healing after surgical repair of rat supraspinatus tendon. They found that FGF-2 administration led to a significant increase in the mechanical strength of repaired tendons at 6 and 12 weeks compared to controls. Additionally, they observed a significant improvement in histological scores at 12 weeks compared to controls, noting more mature, tendon-like tissue at the injury site [31]. Consistent with these findings, Tokunaga et al. found a significantly increased ultimate load-to-failure and stress-to-failure in rabbit supraspinatus tendons treated with FGF-2 12 weeks post-surgery. An improved histological score was seen in the FGF group; the tissue in this group resembled mature tendon tissue, while in the controls, they found loose fibrovascular granulation tissue at the repair site [32]. Another study done in rat supraspinatus tendons shared similar conclusions: FGF appears to enhance tendon healing and maturation [33].

Transforming Growth Factor-Beta (TGF-β)

Transforming growth factor-beta (TGF-β) is involved in promoting chemotaxis and proliferation of tenocytes and fibroblasts, collagen synthesis, and inhibition of fibroblast apoptosis. Similar to IGF, TGF-β levels are elevated through most stages of tendon healing [34]. Furthermore, Kaji et al. found that TGF-β signaling within neonatal tenocytes is necessary for their recruitment to the tendon injury site, suggesting its role in the healing process [35]. However, a link has been found between TGF-β and fibrosis; the effect of this growth factor on tendon healing remains incompletely understood [4,36].

Zhang et al. investigated the effect of a sustained-release TGF-β1 scaffold in a rat model of supraspinatus tendon injury. They demonstrated a dose-dependent increase in tendon maximum load and stiffness at weeks 8 and 16 post-operation. This finding was attributed to increased deposition of ECM primarily containing collagen TIII [37]. This is in contrast to PDGF studies that showed an increase and decrease in collagen type I and III deposition, respectively, with PDGF administration, which was posited to be pro-healing [16,19]. Zhang et al. acknowledge this, explaining that type III collagen synthesis likely enhanced tendon healing indirectly by providing a beneficial ECM microenvironment for fibroblast proliferation [37]. They additionally found that TGF-β1 administration increased expression of IGF-1 and VEGF after surgery, which are posited to enhance tendon healing. This suggests that an endogenous mixture of platelet granule growth factors may provide optimal healing activity in the injured tendon. In another rat supraspinatus sustained TGF-β1 release study, Yoon et al. found that TGF-β1 induced a significant improvement in load-to-failure, ultimate stress, collagen orientation and density, and maturation of tendon to bone, reinforcing the hypothesis that TGF-β plays a positive role in tendon healing [38]. Lastly, Cetik et al. found a significant increase in post-surgery maximum tensile strength with pegylated TGF-β3 treatment in a rat Achilles tendon injury model. They noted a subsequent improvement of histological score in this group, in line with the studies discussed here [39].

Despite demonstrating promising findings, the studies examined in this section utilized acute injury and repair models. Thus, they likely did not reliably replicate the LE pathological microenvironment. Additionally, these studies displayed both positive and inconclusive findings. More work is needed to elucidate the exact effect of each of the growth factors on tendon healing. Nonetheless, the positive effects on tendon healing exhibited by the various treatments discussed suggest their potential to promote tendon healing in patients with refractory LE, which will be examined conjointly, as part of PRP treatment, in the following section.

Current understanding of clinical PRP efficacy

While the definitive effect of the aforementioned growth factors on tendon healing remains incompletely understood, the surrounding evidence supports the claim that they play a role in promoting the tendon healing process. This warrants the examination of the culminated effect of these growth factors as part of platelet alpha granules in PRP treatment, which additionally contain leukocytes, immune system messengers, enzymes, and myriad other bioactive factors that may enhance tendon healing [40]. Exploring the efficacy of this treatment modality is a necessary step in advancing the treatment of LE, as it decreases the risk and cost for patients who have exhausted all other treatment options and are recommended for surgery. Further, it provides a long-term alternative to corticosteroid treatment, which is associated with pain regression, exacerbation of pain due to calcification, and post-treatment rupture and atrophy [41-43]. The existing evidence of PRP treatment efficacy in humans will be examined in order to provide a premise for PRP use in patients with refractory LE.

Myriad studies have explored the efficacy of PRP treatment in improving pain and disability of patients with refractory LE, demonstrating heterogeneous results. Mishra and Pavelko found PRP to result in a 60% improvement in pain scores versus 16% in controls at eight weeks post-treatment. Although no comparison was available between the groups at a mean of 25 months post-treatment, they noted a 93% improvement in pain scores in the PRP group [44]. In another study by Mishra et al., it was demonstrated that there was no difference in pain score improvement between PRP and controls at 12 weeks. However, at 24 weeks, PRP exhibited a significant improvement in pain compared to controls [45]. Along similar lines, Brkljac et al. found that 87.1% of patients treated with PRP showed a minimally clinically important difference in pain scores at a mean follow-up of 5.2 years [46]. In contrast with these findings, Linnanmäki et al. noted no significant difference in pain or disability scores between the PRP group and controls at one-year follow-up [47]. While the data do not clearly point to PRP treatment being beneficial for patients with refractory LE, they suggest its potential in doing so and thus highlight the importance of further work to elucidate the exact role of PRP in the tendon healing process.

PRP Versus Corticosteroids

When all other nonoperative treatments for LE are exhausted, corticosteroids are commonly prescribed. The disadvantage of this treatment is its tendency to diminish in efficacy over time, favoring the use of corticosteroids as a short-term reduction in pain, allowing time to formulate a definitive treatment plan with the aim of eradicating pain. Krogh et al. found that at three months post-treatment, neither PRP nor corticosteroid treatment yielded an improved pain score over saline. They did note that there was a short-term improvement in pain scores at one month in the corticosteroid group [48]. In a similar study, Hohmann et al. demonstrated that corticosteroids are superior to PRP in improving pain scores at one month post-treatment. However, at six months, they found PRP to have a significantly greater improvement in pain scores [49]. In accordance with these findings, Gosens et al. found that while both corticosteroid treatment and PRP treatment improved pain scores at two-year follow-up, the corticosteroid group exhibited a regression in disability scores, unlike PRP, which yielded significantly improved disability scores [50]. These results suggest that steroids are optimal for short-term reduction of pain, creating a buffer time for more definitive treatment options: surgery and, potentially, PRP. Conversely, Shaikh et al. demonstrated no significant difference in pain scores between patients treated with PRP and those treated with corticosteroids [51]. Thus, the data do not clearly indicate PRP treatment to be superior to corticosteroid treatment, although it may suggest it.

PRP Versus Surgery

When corticosteroid efficacy in pain management drops, surgery is the last resort to improve the patient’s condition. While this treatment modality may be beneficial for many patients, it involves general risks of surgery, including but not limited to infection, bleeding, scarring, nerve injury, refractory pain, and the need for further surgery; these risks may be circumvented by replacing surgical intervention with PRP treatment. A study by Boden et al. showed that both ultrasound-guided percutaneous tenotomy and PRP treatment led to significantly improved pain and disability scores [52]. They noted no significant difference between the two treatments. A meta-analysis comparing PRP to surgical intervention of LE by Kim et al. found identical results: PRP and surgery appear to improve pain and disability scores similarly, such that there is no difference between outcomes [53]. Conversely, Merolla et al. found that while both PRP treatment and surgical debridement had similar improvements in the short and medium term, PRP patients exhibited a regression of pain at two years post-treatment [54]. These findings, although not conclusive, suggest the potential for PRP to exhibit similar efficacy to surgical intervention, favoring its use in refractory patients due to its reduced cost and risk of complications.

Leukocyte-rich versus leukocyte-poor PRP

When deciding to utilize PRP therapy for patients with refractory LE, one factor to consider is the leukocyte density of the PRP preparation. From a basic science standpoint, leukocyte-rich PRP (LR-PRP) is thought to have a detrimental effect on the collagen structure of the injured tissue. It has been shown that leukocyte-rich PRP induces an increase in collagen TIII production relative to collagen TI. This is associated with increased fibrosis and poor restoration of tendon mechanical integrity. Conversely, leukocyte-poor PRP (LP-PRP) was shown to increase collagen TI expression relative to TIII, suggesting that LP-PRP may better promote regeneration and not fibrosis [55]. Further, Zhou et al. showed that LR-PRP induces expression of catabolic genes in patellar tendon tenocytes: matrix metalloproteinase-I (MMP-1), MMP-13, tumor necrosis factor-alpha (TNF-α), interleukin-1-beta (IL-1β), and PGE2. In contrast, LP-PRP induced expression of anabolic genes: alpha-smooth muscle actin (α-SMA) and collagen TI/TIII [56]. Despite basic science findings favoring the use of LP-PRP, human clinical studies have not made it clear which preparation optimizes human outcomes. In a meta-analysis by Li et al., they showed that both LP- and LR-PRP treatments led to significantly improved pain and disability scores compared to controls. However, they noted that there was no significant difference in pain relief and functional improvement between LR- and LP-PRP [57]. This conflicts with the findings of a similar meta-analysis by Shim et al., which showed an increase in pain score and success rate only in the LR-PRP group, not the LP-PRP group [58]. Additionally, a direct study investigating the effect of leukocyte density on PRP treatment outcomes found no significant difference in pain score outcomes between the two PRP preparations [59]. Thus, more work is needed to elucidate the role of intra-PRP leukocytes in tendon regeneration to determine the best form of treatment for optimizing patient outcomes.

## Conclusions

In conclusion, lateral epicondylitis is best understood as a degenerative tendinosis characterized by disorganized collagen, reduced cellularity, and increased programmed cell death rather than a persistent inflammatory process. This evolving understanding provides a biologic rationale for the use of platelet-rich plasma, which delivers a concentrated milieu of platelet alpha granule growth factors, including VEGF, PDGF, IGF, FGF, and TGF-β, that have demonstrated pro-healing effects in preclinical tendon models. Animal studies suggest these factors may enhance angiogenesis, tenocyte proliferation, extracellular matrix synthesis, and collagen remodeling, although findings are not uniformly consistent and are largely derived from acute injury models that incompletely replicate the chronic microenvironment of refractory LE. Clinical evidence likewise remains heterogeneous: PRP appears to offer longer-term symptomatic improvement compared to corticosteroids in some studies and may achieve outcomes comparable to surgical intervention, yet other trials demonstrate no significant superiority. Taken together, the current body of evidence suggests that PRP represents a promising biologically based alternative for patients with refractory LE, with potential advantages in durability and safety compared to traditional therapies. However, standardized preparation protocols and long-term randomized studies are necessary to definitively establish its therapeutic role.
